# Preparing for long‐acting PrEP delivery: building on lessons from oral PrEP

**DOI:** 10.1002/jia2.26103

**Published:** 2023-07-13

**Authors:** Connie Celum, Beatriz Grinsztejn, Kenneth Ngure

**Affiliations:** ^1^ Departments of Global Health Medicine, and Epidemiology University of Washington Seattle Washington USA; ^2^ Evandro Chagas National Institute of Infectious Diseases‐Fiocruz Rio de Janeiro Brazil; ^3^ School of Public Health Jomo Kenyatta University of Agriculture and Technology Nairobi Kenya

**Keywords:** adherence, delivery, HIV pre‐exposure prophylaxis, lessons, long‐acting, persistence

## Abstract

**Introduction:**

With recent approvals of long‐acting (LA) HIV pre‐exposure prophylaxis (PrEP) in the form of injectable cabotegravir and the dapivirine ring, programmes need to consider how to optimize the delivery of PrEP methods, including by leveraging lessons from the past decade of oral PrEP delivery.

**Discussion:**

Framed around differentiated service delivery building blocks, the major considerations for the delivery of LA PrEP are how to reach the populations who would most benefit from PrEP, where to locate PrEP services, how to reduce the user burden of accessing and continuing with PrEP, and how to integrate PrEP with other services. Demand creation for LA PrEP and education about new LA PrEP options should be co‐developed with communities and be positively framed. Client‐facing clinical decision support tools provide information about HIV prevention and PrEP options in non‐technical ways and can support their informed decision‐making about PrEP. Training for providers is needed to increase their ability to ask about sexual and drug use behaviours in a non‐judgmental and comfortable manner as part of risk assessment, discuss harm reduction strategies and counsel about available PrEP options that fit clients’ circumstances and needs. PrEP adherence support should include supportive counselling and be tailored to address an individual's particular barriers and needs. Reminders through text messaging or calls can foster PrEP persistence, given the narrow the window around dosing for injectable cabotegravir. Strategies are needed to expand PrEP delivery options, including telePrEP, pharmacy‐based PrEP, key population‐led services and mobile venues. Integrated delivery models are needed which include sexually transmitted infection testing and treatment, contraception for cis‐women not desiring to become pregnant, PrEP for pregnant women in high HIV prevalence settings, and gender‐affirming hormones and support for transgender persons.

**Conclusions:**

The outcome of expanding PrEP options through LA PrEP formulations is to increase PrEP coverage, adherence, persistence and effectiveness by offering a choice of PrEP that meets the needs of persons who would benefit from PrEP. The lessons learned from the delivery of oral PrEP about demand creation, informed client decision‐making, provider training, adherence support and service delivery model are relevant to the delivery of LA PrEP and integration with other services.

## 1 INTRODUCTION

In planning for the implementation of long‐acting (LA) options for HIV pre‐exposure prophylaxis (PrEP), it is important to consider the progress and lessons from the implementation of oral tenofovir‐based PrEP since the U.S. Food and Drug Administration (FDA) approval in 2012. High efficacy was observed in randomized placebo‐controlled trials among men and women who took oral PrEP consistently [1, 2, 3, 4, 5, 6]. Event‐driven oral PrEP has also been demonstrated to have comparable effectiveness to daily oral PrEP among men who have sex with men (MSM), providing an alternative to daily PrEP for this population [7]. Population benefits of oral PrEP have been demonstrated in several settings, including in Australia where following the transition from PrEP delivery from the research context to publicly subsidized care, HIV incidence among MSM remained low over 3 years [8]. In addition, in the context of universal community‐based testing and HIV treatment in the SEARCH trial in Kenya and Uganda, PrEP was offered to persons at elevated risk, one‐third of whom initiated PrEP. Notably, the population HIV incidence in SEARCH communities was reduced by 74%, which demonstrates the population impact of primary HIV prevention in the setting of universal antiretroviral therapy (ART) [9].

Despite impressive levels of protection at an individual and population level, the first decade of PrEP implementation was marked by slow and fragmented implementation with insufficient demand creation, PrEP access, provider training and efforts to reach marginalized key populations. Oral PrEP was not feasible for some users, as healthy persons need to develop the habit of pill‐taking and some have concerns about disclosure and stigma from taking a pill that looks identical to antiretrovirals for HIV treatment. These issues are manifested in the large gap between global targets and the number of people on PrEP. UNAIDs set the target of 3 million PrEP users by 2020, but the estimated number of PrEP users by the end of 2020 was only 1 million, increasing to 2.8 million by mid‐2022 [10]. There is substantial geographic disparity with the majority of PrEP users in North America, Europe and Australia. In 2022, UNAIDS announced a new goal for PrEP of 10 million persons [11]. To achieve a population‐level impact, it will be necessary to improve PrEP persistence and effective use, which are harder to measure than PrEP initiation.

To maximize PrEP impact, innovative solutions are needed that address barriers to PrEP delivery, use, adherence and persistence. Some user barriers to oral PrEP will be addressed by more discrete LA PrEP formulations, specifically bimonthly injectable LA cabotegravir (CAB‐LA) and the monthly dapivirine vaginal ring (DVR), but longer‐acting PrEP formulations alone will not overcome all user barriers. To support LA PrEP implementation, it is useful to review lessons and successful strategies from oral PrEP delivery and consider which strategies are applicable to the delivery of LA PrEP with specific considerations for implementation of DVR and CAB‐LA (Table 1).

**Table 1:** Implementation barriers and strategies to facilitate the delivery of oral and long‐acting PrEP

Barriers to PrEP access, uptake, adherence and persistence	Strategies from oral PrEP that address barriers	Specific considerations for the delivery of dapivirine vaginal ring (DVR)	Specific considerations for the delivery of injectable cabotegravir (CAB‐LA)
**Challenges in reaching populations who could benefit from PrEP**: limited awareness, low perceived risk of HIV, concerns about side effects and use	Demand creation with community input Positive, gain‐framing messages about benefits of PrEP in terms of empowerment and greater intimacy Client‐centred decision support tools Provider training in risk assessment and counselling User and provider PrEP champions to increase knowledge and motivation	Education campaigns about DVR, including safety of DVR during pregnancy and breastfeeding Expand client‐facing tools to describe new PrEP formulations in non‐technical terms in order to provide more informed PrEP decision‐making FAQs about DVR and counselling for providers	Education campaigns about high efficacy and convenience of CAB‐LA, as introduced Expand client‐facing tools to describe new PrEP formulations in non‐technical terms in order to provide more informed PrEP decision‐making FAQs for providers about CAB‐LA dosing, tail and, possible LEVI syndrome
**PrEP delivery**: availability of providers, frequency of visits, stigma from bottles of pills	Reduce frequency of visits through multi‐month dispensing and HIV self‐testing without compromising retention [46]. TelePrEP Pharmacy PrEP programmes Peer PrEP programmes Peer navigators in clinic‐based programmes Reduce out of pocket costs Discrete storage containers Assess substance use, IPV and mental health needs and provide referrals	DVR users would benefit from WhatsApp or text messages for appointment reminders administration Oral fluid HIVST enables less frequent clinic visits Counsel women about discrete storage of DVR and disposal	CAB‐LA is administered every 2 months, with more frequent visits than every 3 months oral PrEP Users would benefit from WhatsApp or text messages for appointment reminders in order to stay in 14‐day window around injections Provider training in giving gluteal injections and private area for administration Oral fluid HIVST may not be sensitive for detecting infections with low antibody levels or during CAB‐LA PK “tail”
**PrEP integration with sexual and reproductive health services**	“One stop services” For women, integration with contraceptive and STI services For TGW, integration with provision of gender‐affirming hormones STI screening and treatment	Counsel women about safety of use of DVR during menses STI screening and treatment	STI screening and treatment Can co‐administer hormonal contraception or gender‐affirming hormones with CAB‐LA Limited drug–drug interactions
**PrEP adherence and persistence**	Menu of adherence support options PrEP support clubs SMS reminders for visits	Reminders for women to replace DVR monthly	Reminders for CAB‐LA injections which are sometimes used for DMPA and NET‐EN could reduce missed visits and the need to restart with a loading dose

Abbreviations: DMPA, depot‐medroxyprogesterone acetate; FAQs, frequently asked questions; HIVST, HIV self‐testing; IPV, intimate partner violence; LEVI syndrome, Long‐acting Early Viral inhibition Syndrom; NET‐EN, norethisterone enanthate: PrEP, pre‐exposure prophylaxis; STI, sexually transmitted infection; TGW, transgender women.

## 2 DISCUSSION

### 2.1 Reaching populations who would benefit from PrEP

One of the biggest challenges with oral PrEP has been reaching populations at greatest need and tailoring PrEP delivery to their circumstances and needs, as manifested in drop‐offs in the PrEP cascade in achieving sufficient uptake, adherence and persistence in order to achieve prevention benefits [12]. A global systematic review found that 41% discontinued with PrEP by 6 months, 47% reinitiated PrEP within 1 year, and with lower discontinuation rates among PrEP projects that included adherence interventions [13]. Those who have a higher risk of not initiating PrEP or premature discontinuation include youth, racial and ethnic minorities, and those without a healthcare provider or without health insurance. The HIV prevention cascade must be strengthened with interventions to stimulate demand from those who would benefit from PrEP, support users in their PrEP adherence and persistence, and reduce health systems barriers [14]. Demand stimulation needs to be motivational and gain‐framed rather than loss‐framed, and co‐developed with communities to find images and messages that motivate potential PrEP users [15]. Although low‐risk perception is cited as a reason why people decline PrEP, using risk calculators and risk‐targeting can themselves increase stigma and be counter‐productive [16]. Other barriers include uncertainty about where to access PrEP, lack of insurance or a provider, costs of PrEP medications and associated testing, concerns about side effects, daily pill‐taking, stigma and competing life priorities [17].

A client‐centred approach to HIV prevention is centred on effectively communicating information about HIV prevention, including the advantages and disadvantages of available HIV prevention methods to facilitate informed decision‐making. The contraceptive field has pioneered patient‐facing decision support tools which have helped facilitate contraceptive choices and increase satisfaction by providing women with an opportunity to compare options and consider their needs and preferences [18]. A PrEP decision support tool needs to include information in non‐technical terms about available PrEP formulations and their effectiveness, safety and user experience in order to facilitate decision‐making about short versus LA PrEP and contraception. A PrEP decision support tool for young African women was modelled after a client‐facing decision support tool for contraception and showed two‐fold higher PrEP persistence at 1 month among women who were randomized to use the tool [19]. Research is needed on how to support integrated PrEP and contraceptive decision‐making and understand the major factors that influence product choices, including side effects, efficacy, convenience and discrete use. More research is needed about whether prospective PrEP users understand the concept of reversibility and whether it is a significant consideration in different populations in their preferences for different PrEP products.

Provider training and engagement are also important, and should include how to ask about sexual and drug use behaviour in an open and non‐judgemental manner, individual's circumstances, need and motivation for PrEP, and specific needs of transgender persons [20]. Providers need to be familiar with available PrEP formulations, counselling about side effects, stopping and restarting PrEP, switching to another PrEP method and HIV testing, including the interpretation and management of atypical HIV test results and HIV RNA screening as part of CAB‐LA delivery [21]. For women, providers need to assess fertility intentions, offer contraceptive options for women who do not want to become pregnant, and counsel about PrEP use and safety during pregnancy and lactation for women.

### 2.2 Expanding PrEP delivery to increase access and acceptability

PrEP implementation for MSM and transgender women (TGW) in Latin America and Asia and adolescent girls and young women (AGYW) in Africa has highlighted the feasibility and benefits of same‐day PrEP delivery and differentiated PrEP service delivery models. Same‐day oral PrEP start was feasible in the ImPrEP study in Brazil, Mexico and Peru [22, 23], which also demonstrated the importance of assessing early adherence to PrEP given that it is associated with PrEP persistence and a lower likelihood of seroconversion [24]. Early follow‐up after PrEP initiation is an opportunity to counsel about side effects, and encourage PrEP adherence and continuation [25]. Additional PrEP adherence support may be beneficial for younger persons, those with less formal education, and those who have experienced discrimination and stigma from healthcare providers. The PrEPARADAS study in Brazil found lower PrEP adherence and persistence among TGW with less education and of black race and higher adherence among TGW receiving gender‐affirming hormones from the study [26].

Community‐based, peer‐led PrEP services for MSM and TGW have been successful. A large number of MSM and TGW were reached through the Princess PrEP programme in Thailand [27], which includes community‐based screening, risk assessment, HIV and transmitted infection STI testing, and PrEP initiation [28]. When CAB‐LA becomes available in national PrEP programmes, peers could offer CAB‐LA or oral PrEP, although the gluteal CAB‐LA injection would need to be administered by a healthcare professional, requiring close linkages with healthcare facilities. Task‐shifting of PrEP prescribing from physicians to nurses has significantly increased PrEP prescribing in Brazil [29]. Providers who are PrEP champions are very effective in motivating and supporting other providers during PrEP implementation.

Diversification of PrEP delivery points and task shifting are also needed to expand PrEP coverage in Africa. PrEP was initially delivered in HIV clinics, which worked well to increase PrEP uptake among HIV‐uninfected partners in HIV serodifferent couples through integrated care programmes delivering both ART and PrEP [30, 31]. Increasingly, PrEP is being integrated into other service points to cater for different populations, such as in antenatal clinics [32, 33], family planning clinics [33], post‐abortion care [34] and STI clinics [35].

One population that has lagged behind in PrEP effective use is African AGYW due to low adherence and persistence; multiple PrEP demonstration projects have shown encouragingly high PrEP uptake among African AGYW but lower adherence and persistence than MSM [36]. Mobile adolescent‐friendly health services have successfully reached South African AGYW for PrEP, contraception, and sexually transmitted infection (STI) testing and treatment [37]. The high efficacy and bimonthly dosing of CAB‐LA could address challenges with adherence and persistence of oral PrEP among African AGYW, many of whom use injectable contraception, but will involve bimonthly instead of quarterly visits, which need to occur within a 14‐day window to maintain sufficient cabotegravir levels.

Pharmacy‐based PrEP delivery is another innovation to bring PrEP closer to potential users and to avoid clinic waits and other barriers, which could and expand to include the delivery of LA PrEP [38, 39, 40, 41]. In Vietnam, differentiated PrEP delivery has supported increased scale‐up; one‐third of clients initiated PrEP at clinics led by community health workers who are MSM or TGW and PrEP is now being rolled out in pharmacies [42]. Characteristics of PrEP initiators and data about PrEP persistence from different service points and providers will provide useful insights for LA PrEP delivery.

Another method to increase PrEP persistence is to improve efficiency during clinic visits. The promising “one stop” PrEP delivery model could be expanded to include LA PrEP as another PrEP option [43]. Laboratory screening for PrEP should be streamlined. Many PrEP programmes have shifted to offering same‐day PrEP after a negative HIV test, given the rarity of renal insufficiency in young populations [44]. Same‐day PrEP starts are feasible with the DVR but will be more challenging with injectable PrEP, given the turnaround time for viral load testing, and the need for prior authorization for insurance approvals in settings such as the U.S. Laboratory monitoring can be a cost and logistic barrier to PrEP implementation and should be carefully considered. Notably, Kenyan guidelines for oral PrEP include optional renal monitoring given the rarity of nephrotoxicity [45]. After PrEP initiation, quarterly HIV testing is recommended, which typically necessitates clinic visits. Encouragingly, a recent study in Kenya demonstrated that HIV self‐testing is acceptable to PrEP users and reduced the frequency of clinic visits from every 3 to every 6 months without compromising PrEP retention, adherence and HIV testing [46]. Secondary distribution of HIV self‐test kits could also increase partner testing and identify prospective PrEP users [47]. However, CAB‐LA PrEP requires every 2‐month visits for injections, and may require viral load monitoring to detect seronegative early HIV infection [48]; the more frequent visit schedule could be burdensome for users and viral load testing adds to the costs of CAB‐LA [49].

### 2.3 Achieving adherence with PrEP

Adherence to oral PrEP has been challenging for some populations, such as African AGYW, in part due to stigma from tablets and bottles that look identical to antiretrovirals for treatment, the need to establish pill‐taking habits, and the dynamic nature of sexual behaviour and perceived HIV risk. Strategies to support oral PrEP adherence include retrospective drug‐level feedback based on intracellular tenofovir diphosphate levels; however, the testing is costly and complex to implement and did not increase PrEP adherence among African AGYW [50, 51]. Low‐cost, same‐day point‐of‐care urine assays that measure tenofovir use in the past 5 days are promising for adherence counselling [52]. Peer support, more frequent visits and flexible adherence support interventions may address challenges for African AGYW [49]. Given low adherence to oral PrEP and DVR among African AGYW, the REACH crossover study of oral PrEP and the DVR among late adolescent girls ages 16–21 had encouraging findings with over half having high adherence to both oral PrEP and DVR in the context of monthly visits and a menu of adherence support options [53].

The goal is for high PrEP adherence during periods when an individual has a greater risk of HIV exposure (“prevention‐effective adherence”) [54, 55], which highlights the need for periodic reassessment of risk after PrEP initiation. A PrEP demonstration project among AGYW in southern Africa found evidence of prevention‐effective adherence and higher PrEP adherence during periods of higher risk [56]. Providers need to be aware that although cycling on and off PrEP is expected, the high HIV incidence observed after PrEP discontinuation indicates the need for ongoing risk assessment to identify individuals who have ongoing risk and for whom stopping PrEP can be premature [57].

### 2.4 Integrating PrEP with other reproductive and sexual health services

Given that the populations who have the highest HIV incidence rates and the greatest need for PrEP often are also at risk for bacterial STIs, PrEP delivery should include STI testing and treatment. STI rates are very high with a pooled incidence of chlamydia, gonorrhoea and early syphilis of 72.2 per 100 person‐years across diverse geographies and populations taking PrEP [58]. In 2022, the WHO provided guidance on the integration of STI services into PrEP programmes [59].

Sexual and reproductive health services need to be integrated into PrEP delivery for cis‐gender women, with an assessment of fertility intentions, provision of contraception options for women who desire to not become pregnant, and screening and referral for intimate partner violence (IPV) [60]. Studies have reported that IPV interferences with women's interest and willingness to use PrEP, and is associated with interruptions in PrEP use, and poor adherence [60]. Symptoms of depression have been prevalent among African AGYW in PrEP demonstration projects with persistent depressive symptoms associated with lower PrEP adherence and persistence [61]. LA PrEP products that do not require daily adherence may be a good option for persons experiencing depression or IPV.

For transgender populations, PrEP provision in gender‐affirming environments improves uptake and adherence and enhances the effectiveness of PrEP delivery [62, 63]. Gender‐affirming hormone therapy (GAHT) is a key aspect of the standard of care for TGW to induce secondary female sex characteristics while reducing male sex characteristics. Key components of gender‐affirming and HIV prevention primary care include providing adequate information for TGW on the lack of drug–drug interactions between PrEP and GAHT which may increase PrEP adherence, capacity building and training for key population‐led clinics, one‐stop primary healthcare for transgender populations, and national HIV and transgender healthcare guidelines. Thigh injections of CAB‐LA are feasible, well‐tolerated and provide an injection option for persons with gluteal implants [64]. Lastly, stimulant use and chemsex is prevalent among MSM and transgender populations in PrEP programmes [65, 66], and presents additional challenges with PrEP adherence and persistence. If PrEP programmes are not able to provide substance use interventions, referrals should be provided.

## 3 CONCLUSIONS

Experiences from oral PrEP implementation have identified strategies to mitigate user and provider barriers which have relevance to the introduction of new LA PrEP formulations, namely DVR and CAB‐LA, which reduce user burden associated with oral PrEP, and could improve coverage through increasing choice to allow users to identify which PrEP option best fits their needs. However, new products have costs and programmatic demands, such as supply chain management that will impact their introduction. Demonstration projects are useful to identify the best practices of how to implement new PrEP products, and it may be beneficial to stagger the introduction of new PrEP products in order to support uptake (e.g. through decision support tools for clients and training of providers) and digital tools for visit reminders in order to foster PrEP persistence. In addition, structural factors, such as poverty, stigma and access barriers to HIV prevention, disproportionately impact vulnerable and often marginalized populations. Strategies to address these inequities need to be a focus of LA PrEP implementation, so that access gaps to these exciting new PrEP formulations do not widen. The past decade of oral PrEP implementation highlights the need to move from facility‐based, medicalized models of HIV prevention, to provide non‐stigmatizing and supportive counselling, and to deliver PrEP as part of an integrated service package. Programmes need to partner with communities in demand creation and delivery models, learn from users about what they need to support them in PrEP choice and continuation, and support providers to ensure that the second decade delivers the promise of LA PrEP, in part by building on lessons from the first decade of implementing oral PrEP.

## COMPETING INTERESTS

CC has received research grants from NIH, USAID, and Bill and Melinda Gates Foundation, has served on the advisory board for Gilead Sciences and Merck, and has served as an expert witness for Gilead. BG has served on advisory boards for Janssen, GlaxoSmithKline and Merck. KN has received research grants from NIH, Bill and Melinda Gates Foundation, and Merck.

## AUTHORS’ CONTRIBUTIONS

CC prepared the initial draft of the manuscript, and BG and KN revised the manuscript. All authors have read and approved the final manuscript.
